# Targeting the NAT10/XIST/YAP1 Axis-Mediated Vascular Abnormalization Enhances Immune Checkpoint Blockade in Gastric Cancer

**DOI:** 10.7150/ijbs.113325

**Published:** 2025-07-28

**Authors:** Xuetao Lei, Boyang Zheng, Yanmei Peng, Guofan Zhang, Xia Cheng, Wenqiang Li, Jiayong He, Fengping Li, Ruoyu Ling, Ziyi Fu, Qingbin Yang, Gengtai Ye, Guoxin Li

**Affiliations:** 1 Department of General Surgery & Nanfang Gastrointestinal Cancer Institute (NGCI), Nanfang Hospital, Southern Medical University, Guangzhou, Guangdong 510515, P. R. China.; 2 The First School of Clinical Medicine, Southern Medical University, Guangzhou, Guangdong 510515, P. R. China.; 3 Guangdong Provincial Key Laboratory of Precision Medicine for Gastrointestinal Tumor & Guangdong Provincial Engineering Technology Research Center of Minimally Invasive Surgery, Guangzhou, Guangdong 510515, P. R. China.; 4 Cancer Center of Beijing Tsinghua Changgung Hospital, School of Clinical Medicine, Tsinghua Medicine, Tsinghua University, Beijing 102218, P. R. China.; 5 Department of Gastrointestinal Surgery, The First Affiliated Hospital of Guangzhou Medical University, Guangzhou, Guangdong 510120, P. R. China.; 6 Department of breast surgery, Affiliated Hospital of Guangdong Medical University, Zhanjiang, Guangdong 524002, P. R. China.; 7 Zhanjiang Key Laboratory of Intelligent Diagnosis and Treatment of Breast Cancer, Zhanjiang, Guangdong 524002, P. R. China.; 8 Department of General Surgery, Aerospace Center Hospital, Beijing 100049, P. R. China.

**Keywords:** NAT10, XIST, gastric cancer, vascular normalization, YAP1

## Abstract

Tumor vascular normalization has emerged as a promising strategy to potentiate immune checkpoint blockade in solid tumors. Here, we unveil a previously unrecognized NAT10/XIST/YAP1/VEGFA signaling axis driving vascular abnormalization in gastric cancer (GC) and demonstrate its therapeutic potential in remodeling the tumor immune microenvironment. Through integrative analysis of acetylated RNA immunoprecipitation sequencing (acRIP-seq) and functional validation, we identified NAT10-mediated N4-acetylcytidine (ac4C) modification as a critical stabilizer of lncRNA XIST. Mechanistically, XIST recruits hnRNPK to facilitate YAP1 nuclear translocation, thereby activating TEAD4-dependent VEGFA transcription and promoting angiogenic programming. Genetic or pharmacological inhibition of NAT10 with Remodelin attenuated VEGFA secretion, enhanced pericyte coverage and basement membrane integrity, and normalized tumor vasculature in syngeneic GC models. Moreover, we found that NAT10 inhibition reshaped the immune landscape by upregulating CXCL9/10/11 chemokines, promoting cytotoxic lymphocyte infiltration while reducing Treg populations. Strikingly, combining Remodelin with the YAP1 inhibitor Verteporfin synergistically augmented anti-PD-1 efficacy, significantly suppressing tumor growth in immunocompetent mouse models. Our findings not only elucidate an ac4C-dependent epitranscriptomic mechanism governing vascular-immune crosstalk but also propose a novel combinatorial therapeutic strategy to overcome resistance to immune checkpoint blockade in GC.

## Background

The efficacy of cancer immunotherapy critically depends on dynamic crosstalk between malignant cells and stromal components within the tumor microenvironment (TME), particularly immune effector cells^1^, ^2^. While these interactions are well-characterized, emerging evidence highlights the pivotal role of vascular networks in modulating therapeutic outcomes^1^, ^2^. Tumor-associated angiogenesis, a hallmark of malignancy, generates structurally abnormal vasculature characterized by tortuous, hyperpermeable vessels. This vascular dysfunction elevates interstitial fluid pressure, exacerbates hypoxia, and fosters an immunosuppressive TME marked by diminished CD8+ T-cell infiltration and enrichment of myeloid-derived suppressor cells (MDSCs), M2-polarized tumor-associated macrophages (TAMs), and regulatory T cells (Tregs)^1^, ^3^, ^4^. Such pathological angiogenesis is driven by the accumulation of pro-angiogenic factors including vascular endothelial growth factor (VEGF), fibroblast growth factor (FGF), transforming growth factor-β (TGF-β), and platelet-derived growth factor (PDGF) within the TME^1^, ^2^. Notably, VEGF-A, a hypoxia-inducible angiogenic master regulator, has been shown to synergize with PD-1 blockade when targeted therapeutically^5^.

Epigenetic modulation has emerged as a promising strategy to potentiate immunotherapy in tumor patients^6^, ^7^ . Beyond canonical epigenetic mechanisms (DNA methylation, histone modifications, N6-methyladenosine (m6A) m6A RNA methylation), the recently characterized N4-acetylcytidine (ac4C) RNA modification plays critical roles in antiviral immunity and tumor immune evasion^8^-^10^. N-acetyltransferase 10 (NAT10), the sole known ac4C "writer" enzyme, promotes Treg infiltration through metabolic reprogramming of tumor glycolysis and lactate secretion^10^, ^11^. While NAT10 promotes tumor proliferation, migration, invasion and cisplatin chemoresistance via mRNA ac4C modification^10^-^16^, its regulation of non-coding RNA acetylation (e.g., lncRNAs, circRNAs) and vascular remodeling remains unexplored^17^.

Here, we demonstrate that NAT10 drives pathological angiogenesis and vascular dysfunction in murine tumor models. Mechanistically, NAT10 acetylates lncRNA XIST to facilitate hnRNPK-mediated nuclear translocation of Yes-associated protein 1 (YAP1), thereby activating YAP1-dependent VEGFA transcription under both normoxic and hypoxic conditions. Therapeutic inhibition of NAT10 with Remodelin normalized tumor vasculature, reduced interstitial pressure, and enhanced CD8+ T-cell infiltration through CXCL9/10/11 upregulation. In immunocompetent mouse models, Remodelin synergized with YAP1 inhibition (Verteporfin) to potentiate anti-PD-1 checkpoint blockade, establishing dual NAT10/YAP1 targeting as a strategy to overcome vascular-mediated immunotherapy resistance.

## Materials and Methods

### Clinical samples and cell lines

Paired gastric cancer (GC) tissues and histologically normal adjacent gastric mucosa specimens were obtained from treatment-naïve patients undergoing curative resection at Nanfang Hospital, Southern Medical University. All participants provided written informed consent, and this study was approved by the Ethics Committee of Nanfang Hospital (Guangzhou, China).

The human gastric cell lines (AGS and MGC-803) and HEK-293T were purchased from the Cell Bank of Chinese Academy of Sciences (Shanghai, China). The mouse forestomach carcinoma (MFC) cell line was purchased from National Cell Resource Center, Peking Union Medical College (PCRC). The human umbilical vein endothelial cells (HUVECs) were ordered from American Type Culture Collection (ATCC). All the cell lines were cultured in Dulbecco's Modified Eagle Medium (DMEM; Gibco) containing 10% fetal bovine serum (FBS, BioInd, Israel) and 1% Penicillin-Streptomycin 37℃ in a humidified atmosphere of 5% CO_2_. Cobalt chloride (CoCl_2_; Sigma-Aldrich, St Louis, MO, USA) was used to simulate hypoxic conditions as previously reported^11^. Briefly, cells were treated with 100μM CoCl_2_ and incubated for 8-24h to induce hypoxia-mimetic responses.

### Experimental animal models

To establish syngeneic tumor models, female 615 mice (6-8 weeks old; Institute of Hematology, Chinese Academy of Medical Sciences) received subcutaneous implantation of 6×10⁵ MFC cells suspended in 100 μL PBS into the right flank. For xenograft experiments, athymic BALB/c nude mice (4-5 weeks old; Central Laboratory of Animal Science, Southern Medical University, Guangzhou) maintained under specific pathogen-free conditions received analogous injections with 6×10⁵ human gastric cancer (GC) cells. Pharmacological interventions included: 1) Remodelin (NAT10 inhibitor, 100 mg/kg; Selleck #S7641) formulated in DMSO and administered via oral gavage every 48 hours; 2) Anti-PD-1 monoclonal antibody (200 μg/mouse, MedChemExpress, #HY-P99144) reconstituted in 50 μL saline for intraperitoneal injection every 72 hours; and 3) Verteporfin (YAP1 inhibitor, 100 mg/kg; Selleck #S1786) dissolved in PBS and delivered intraperitoneally on alternate days. Tumor dimensions were serially monitored every 5 days using digital calipers, with volume calculated as (major axis × minor axis²)/2. Terminal tumor specimens were collected, fixed in 4% paraformaldehyde (PFA), and processed for histopathological evaluation.

### Lentiviral transduction and siRNA transfection

Recombinant lentiviral vectors (Genecopoeia) were packaged using the Lenti-Pac HIV Expression System (Cat# LT002) following manufacturer's specifications. For TEAD isoform knockdown, human gastric cancer cells were transfected with TEAD1-4-specific siRNAs (Ribobio, China) complexed with Lipofectamine 3000 (Invitrogen, #L3000015) at 60-70% confluency. All plasmid constructs (see Table S1) underwent endotoxin-free purification and Sanger sequencing validation prior to use.

### Immunofluorescence (IF) and fluorescence in situ hybridization (FISH) microscopy

For paraffin section immunofluorescence, fresh specimens were fixed in 4% paraformaldehyde (24h, 4°C), dehydrated through ethanol-xylene series, and paraffin-embedded. Sections (4 μm) were deparaffinized, rehydrated, and subjected to epitope unmasking via pressurized heating in citrate buffer (pH 6.0, 15 min). After permeabilization with 0.3% Triton X-100/PBS (20 min), sections were blocked with 5% BSA/0.1% Tween-20 (1h, RT) and incubated with primary antibodies for 16 h (4°C, Table S3). After washing, the sections were incubated with fluorescent secondary antibodies (Table S3) for 1 hour at room temperature, and counterstained with DAPI for 10 min. Images were taken by confocal laser scanning microscopy and analyzed using SlideBook software and ImageJ.

For cell IF staining, cells were plated on confocal microscope plates (NEST) and incubated overnight. The cells were then fixed with 4% paraformaldehyde for 15min and permeabilized with 0.1% Triton X-100 in PBS for 10min. After blocking with 5% BSA for 30min at 37°C, the cells were incubated with anti-YAP1 antibody overnight at 4°C. Next day, the cells were stained with the corresponding fluorescent secondary antibodies and the nuclei were stained with 4′,6-diamidino-2-phenylindole (DAPI). Fluorescence images were acquired by a confocal laser scanning microscope (LSM980, Zeiss).

For FISH, cells were fixed with 4% paraformaldehyde for 10 min at room temperature. For the detection of XIST, RNA FISH was performed with a Fluorescence in Situ Hybridization Kit (D-2968, Exon Biotechnology Inc, Guangzhou, China) following the manufacturer's instructions. The has-XIST ISH probe (Dig, 007.254210) were designed by Exon Biotechnology Inc. To analyze the colocalization between lncRNA XIST and hnRNPK protein, we added anti-hnRNPK antibody while incubating cells with anti-digoxigenin antibody, and used red fluorescent secondary antibody to visualize XIST and green fluorescent secondary antibody to visualize hnRNPK according to species differences. Images were acquired by confocal laser scanning microscopy (Zeiss, Jena, Germany).

### Hypoxia, perfusion, and vascular leakage measurements

Tumor-bearing mice were intraperitoneally injected with Hypoxyprobe-1 (40mg/kg, Hypoxyprobe kit) 1h before sacrifice to assess tumor hypoxia. To detect the formation of pimonidazole adducts, tumor frozen sections were stained with FITC-labeled mouse anti-pimonidazole monoclonal antibody following the manufacturer's instructions. To assess vascular leakage, 5mg of FITC-labeled dextran (40kDa; FD40S Sigma-Aldrich) was intravenously injected and the mice were sacrificed to harvest tumors after 30min. Cryosections were made for downstream IF staining and quantification. Tumor perfusion was quantified on tumor cryosections after intravenous injection of FITC-conjugated Tomato lectin (1mg/mL, 100μL, Vector Laboratories) into tumor-bearing mice 10min before tumors were harvested for analysis. The perfused tumor vasculature was defined as FITC+ CD31+ vessels.

### Chick embryo chorioallantoic membrane (CAM) assay

Fertilized chicken eggs were incubated at 37.8°C with 60-70% relative humidity. After cutting a round window into an egg, 30μL of cell culture supernatant was dropped onto a filter paper disc and sealed with transparent tape. On the day 10, the eggs were photo-graphed with a MacroPATH dissecting microscope (Milestone, Italy), and the number of blood vessels around the filter paper disc was counted.

### Endothelial tube formation analysis

The HUVECs were cultured in tumor supernatants from each cell line in 96-well plates at a density of 1 × 10^4^ cells per well for 6 h. Plates were precoated with 50μL Matrigel (R&D Systems, 3533-010-02) at 37°C for 1h. After a 6h incubation, tubule images were acquired and analyzed by Image-Pro Plus software, which we quantified by counting the number of cell junctions and tubes in 10 randomly chosen fields of view. Data were obtained from three independent experiments.

### Transwell cell invasion assay

For the invasion assay, the Transwell chamber was coated with Matrigel (Corning, 356234). The HUVECs were suspended in serum-free medium (1×10^5^ cells/insert) and added to the upper chamber of the 24-well insert, and the lower chamber was filled with 600μL of complete medium. After incubation for 12h, the cells migrated to the bottom of the membranes were fixed and stained with 0.1% crystal violet for 30min.

### Wound healing assay

The HUVECs were seeded into a 6-well and incubated to a reach confluence. The monolayer was scratched using a 200μL sterile plastic tip and the detached cells were removed with PBS. Then the HUVECs were cultured with the conditioned medium (CM) from GC cells. The images were taken at 0h, 12h, and 24h. Each assay was replicated three times.

### RNA isolation and qRT-PCR

We used RNA-Quick Purification Kit (ES Science, Shanghai, China) to isolate total RNA from samples. cDNA was synthesized from 1 μg of total RNA using PrimeScript RT Master Mix (RR036A, Takara, Japan). SYBR Green qPCR Master Mix (RR420, Takara, Japan) was used for the qRT-PCR analysis according to the manufacturer's instructions. The designed primers for the qPCR are listed in Table S2.

### Western blot analysis and Enzyme-linked immune-sorbent assay (ELISA)

Proteins were extracted using RIPA buffer supplemented with protease and phosphatase inhibitors (Beyotime, China) and quantified with a BCA kit. The proteins were then separated via sodium dodecyl sulfate‒polyacrylamide gel electrophoresis (SDS-PAGE) and transferred to PVDF membranes (Millipore, Massachusetts, USA). Subsequently, the membranes were blocked in 5% nonfat milk for 1h and incubated with a primary antibody at 4°C overnight. The membranes were hybridized with secondary antibodies for 1h at room temperature. An enhanced chemiluminescence (ECL) substrate kit (WBKL0100, Millipore) was then used to visualize the membrane. Moreover, the human VEGFA ELISA Kit (RAB0507, Sigma-Aldrich) and human Angiopoietin-2 Quantikine ELISA Kit (DANG20, R&D Systems) were used to detect the cell culture medium according to the manufacturer's instructions.

### RNA decay assay

GC cells were treated with the RNA transcription inhibitor actinomycin D (5µg/mL) for 0, 4, 8 ,12, 16h. Then, the cells were collected for total RNA extraction. qRT‒PCR was used to quantify the relative abundance of XIST lncRNA (relative to 0h).

### Nuclear and cytoplasmic extraction

Cells were lysed to isolate the nuclear and cytoplasmic fractions using the Nuclear and Cytoplasmic Protein Extraction Kit (Beyotime, Shanghai, China) according to the manufacturer's instructions. The samples were separated by SDS-PAGE and immunoblotted for Tubulin and Laminb1 as cytoplasmic and nuclear markers, respectively.

### Acetylated RNA immunoprecipitation (acRIP)

To quantify the level of ac4C modification of the specific gene, acetylated RNA immunoprecipitation (acRIP) was performed using an EpiTM ac4C immunoprecipitation kit (Epibiotek Co., Ltd.) following the manufacturer's instructions. Briefly, fragmented total RNA was subjected to magnetic immunoprecipitation with the anti-ac4C antibody at 4°C overnight. After the RNA was eluted from the beads, qPCR was performed.

### RNA immunoprecipitation (RIP) assay

An RNA immunoprecipitation (RIP) kit (Bes5101, Bersinbio, China) was used according to the manufacturer's instructions. Briefly, cells were lysed in polysome lysis buffer containing protease inhibitor and RNase inhibitor. The cell lysate was incubated with anti-immunoglobulin G (IgG) or the indicated antibody overnight at 4°C. Then, RIP buffer containing magnetic beads was incubated with the cell lysate for 1h. To isolate the immunoprecipitated RNA, the samples were incubated with proteinase K digestion buffer. Finally, the purified RNA was subjected to qPCR to confirm the presence of the binding targets.

### RNA pulldown

The RNA pulldown assay was conducted using the RNA-Protein Pull-Down Kit (Pierce, USA) following the manufacturer's protocol. Firstly, we used the RiboMAX™ Large Scale RNA Production Systems (Promega, USA) to transcribe full-length XIST and labeled it with biotin using the Biotin RNA Labeling Mix (Roche, Switzerland). The biotin-labeled XIST probe incubated with streptavidin magnetic beads for 60min at room temperature. After being bound to streptavidin magnetic beads, the probe-bead complexes were incubated with whole GC cell lysates overnight at 4°C. After separation and purification, the eluate was subjected to Western blot analysis.

### Luciferase reporter assay

To evaluate the effect of the ac4C peak on XIST lncRNA stability, the wild-type or mutant ac4C peak region of XIST was inserted into reporter plasmids. The luciferase reporter assay was performed following the protocol of the Dual-Luciferase Reporter Kit (Cat# LF004, GeneCopoeia). Upon NAT10 knockdown or overexpression, firefly and Renilla luciferase activities were quantified using a Dual-Luciferase Reporter Assay System (Promega, Madison, WI, USA) in a ModulusTM II Microplate Multimode Reader (Promega) according to the manufacturer's protocols. Firefly luciferase (FLuc) activity was measured and normalized to the Renilla luciferase (Rluc) activity. The experiments were carried out in triplicate.

### Chromatin immunoprecipitation (ChIP) assay

The ChIP assay was performed using the PierceTM Agarose ChIP kit (Pierce; Thermo Scientific, Rockford, IL, USA) according to the manufacturer's protocol. Immunoprecipitation was carried out using rabbit polyclonal TEAD4 antibody (Proteintech, 12418-1-AP). An unrelated rabbit IgG was used as a negative control. To amplify the potential TEAD4-binding site from nucleotides -1452 to -1443 in the promoter of VEGFA, qPCR was performed using the forward primer 5'-AGGGCAGAATCATCACGAAGT-3' and the reverse primer 5'-AGGGTCTCGATTGGATGGCA-3'. The anti-RNA polymerase II (anti-RNAPII) antibody and GAPDH qPCR primers were provided as a positive control in the kit for assay technique and reagent integrity. Data analysis was performed using the 2^ (-ΔΔCt [ChIP/NIS])^ method.

### Flow cytometry

Tumor tissues were collected and digested in DMEM containing collagenase type1A, hyaluronidase, and DNase I to prepare a single-cell suspension. Cells were washed in cold flow buffer (0.1% NaN3 in PBS +1% bovine serum albumin). The fluorochrome coupled antibodies against different T cell were purchased from BioLegend. Dead cells were excluded with 7-Amino-actinomycin D (7AAD) (eBioscience). Flow cytometric analysis was conducted with BD FASC Aria III, and data were analyzed using FlowJo software (Tree Star Inc.).

### Statistical analysis

All quantitative data are presented as mean ± standard deviation (SD) unless otherwise specified. Statistical analyses were performed using GraphPad Prism 8.0 (GraphPad Software). Between-group comparisons were analyzed by unpaired two-tailed Student's t-test. For multi-group comparisons, one-way ANOVA (single independent variable) or two-way ANOVA (two independent variables) was conducted with Bonferroni post hoc testing. Statistical significance was defined as *p < 0.05, **p < 0.01, and ***p < 0.001.

## Results

### NAT10 inhibition disrupts angiogenic programming and induces vascular normalization in gastric cancer

**Figure 1:**
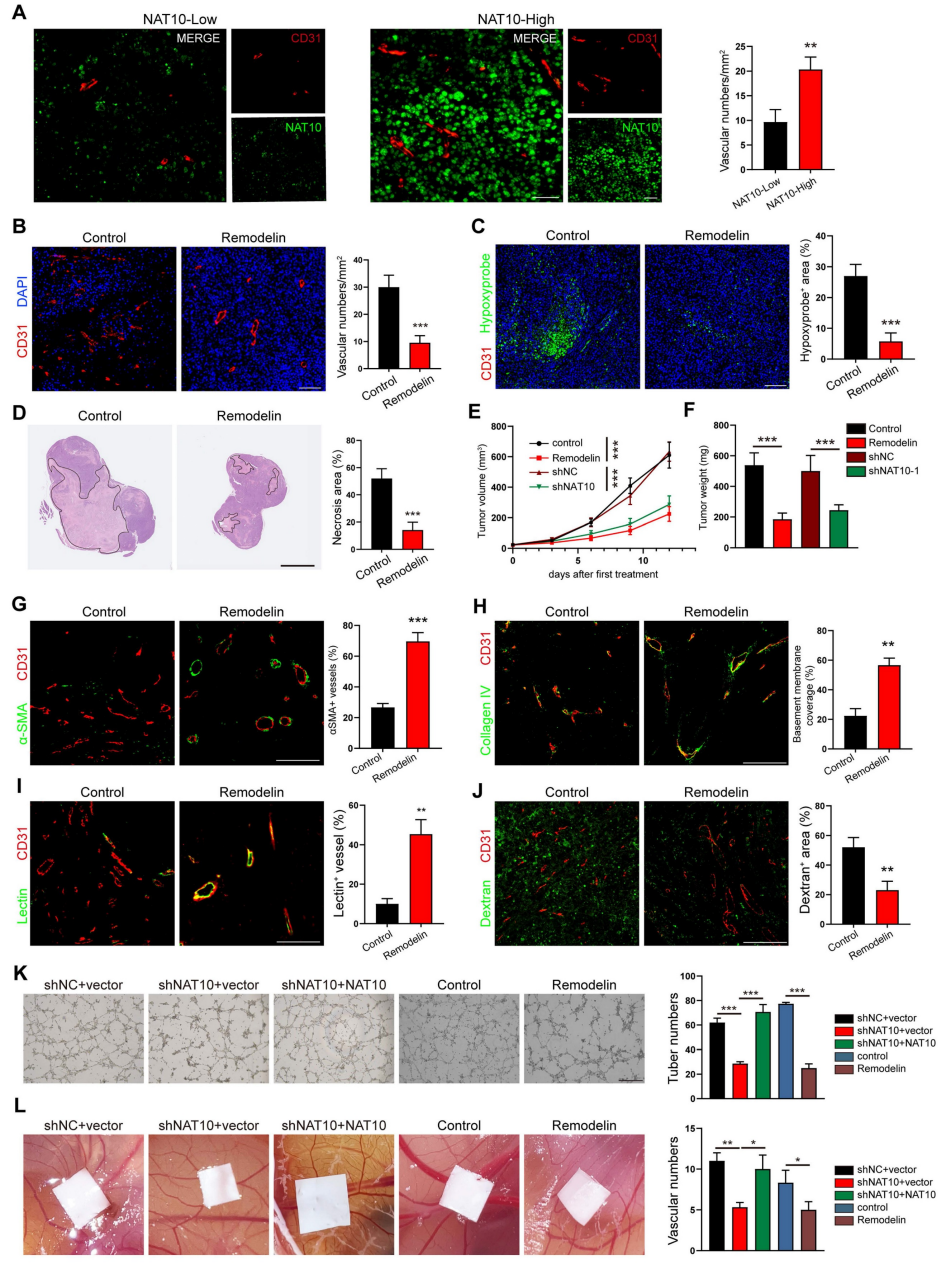
Remodelin impairs NAT10-mediated angiogenesis and promotes vascular normalization in GC. (A) The expression of NAT10 (green), and the number of vessels (CD31, red) in tumors from GC patients were detected by IF assays. Scale bar: 50 μm. (B) The blood vessel numbers within MFC tumors treated with Remodelin were quantified by CD31 staining. Scale bar: 50 μm. (C) Representative images of Hypoxyprobe-1-labeled areas in MFC tumors treated with Remodelin. Scale bar: 50 μm. (D) Representative images of MFC tumor necrosis. The H&E staining was conducted and the necrosis area was labeled using lines. Scale bar: 2.5 mm. (E, F) The volume (E) and weight (F) of MFC tumors in mice were analyzed by 2-way ANOVA. (G, H) IF images of αSMA (G), and Collagen IV (H) in MFC tumors. The pericyte coverage and BM coverage were measured by using ImageJ software. Scale bar: 50 μm. (I, J) Representative images for vessels perfused with lectin (I) and dextran (J) in MFC tumors treating with Remodelin. Scale bar: 50 μm. (K) The tube formation abilities of HUVECs treated with CM from AGS cells transfected with NAT10 shRNA plasmids or treated with Remodelin were analyzed. Scale bar: 500 μm. (L) The CAM assay was performed to investigate the effect of CM from AGS cells transfected with NAT10 shRNA plasmids or treatment with Remodelin.

Building upon our prior findings demonstrating elevated NAT10 expression in central tumor (CT) regions compared to invasive margins (IM)^11^, subsequent spatial analysis revealed distinct vascular patterning differences. Quantitative immunofluorescence mapping showed significantly increased CD31+ microvessel density in CT areas versus IM regions (Fig. 1A), suggesting paradoxical hypervascularization despite hypoxic tumor cores. Notably, these CT-associated vessels exhibited impaired maturation markers: 1) Pericyte coverage: The α-SMA+ vascular investment decreased by more than 60% in CT vs IM (*p* < 0.001); 2) Basement membrane integrity: The collagen IV deposition showed approximately 1-fold reduction in CT areas (*p* < 0.01) (Figure S1A, B). Our findings position NAT10 as a potential regulator of vascular normalization processes in GC progression.

To further confirm the positive correlation of NAT10 in GC cells with increased tumor vessel density, we established two types of models: (1) Model 1, in which the NAT10-specific inhibitor Remodelin was used to treat syngeneic subcutaneous GC models, which were established by subcutaneously injecting mouse forestomach carcinoma (MFC) cells into 615 mice; (2) Model 2, in which we generated syngeneic subcutaneous GC models by implanting shNat10 MFC cells and their negative control cells. In both of these models, we harvested tumors and analyzed the vascular density, tumor cell hypoxia and necrosis. The CD31 staining revealed a significant reduction in microvessel density in both Remodelin-treated and shNat10 mice (Figure 1B and Figure S1C). Moreover, we observed that inhibiting NAT10 with Remodelin or shRNA resulted in a significant reduction in hypoxic areas and cell necrosis within tumors (Figure 1C, D and Figure S1D, E), and tumor volumes and weights were effectively reduced compared to their respective negative controls (Figure 1E, F). Notably, we found that tumor vessels from the Remodelin-treated group exhibited increased pericyte coverage (Figure 1G) and enhanced BM coverage (Figure 1H), indicating that Remodelin facilitated the normalization of tumor blood vessels. To investigate whether structural changes in the tumor vasculature following NAT10 inhibition could lead to functional enhancements, mice were intravenously injected with fluorescein isothiocyanate-lectin (FITC-lectin) or FITC-dextran. We found that the number of abnormal blood vessels, characterized by poor vessel perfusion and hyperpermeability, was significantly reduced in the Remodelin-treated mouse tumors (Figure 1I, J). Consistently, more mature and stable tumor vessels were also observed in shNat10 tumors (Figure S2F-I). Thus, NAT10 inhibition in GC cells promoted tumor vascular normalization.

To further investigate whether the expression of NAT10 in GC cells promotes tumor angiogenesis, we used the conditioned medium (CM) from NAT10 knockdown (shNAT10) or NAT10-inhibited (Remodelin) GC cells to cultivate HUVECs. We found that the tube formation ability of HUVECs in the NAT10-knockdown or NAT10-inhibited group was significantly decreased (Figure 1K). Similarly, the migration and invasion capabilities of HUVECs markedly decreased in the NAT10-knockdown or Remodelin treatment groups (Figure S2A, B). As expected, the tubule formation, migration and invasion abilities of HUVECs were restored by re-expressing NAT10 (Figure 1K and Figure S2A, B). Furthermore, the results of the chick embryo chorioallantoic membrane (CAM) assay showed that treatment with CM from NAT10-knockdown or inhibited GC cells significantly reduced the vessel number (Figure 1L), whereas the CM from NAT10-reexpressing GC cells restored vessel formation (Figure 1L). These results indicated NAT10 as a master regulator of GC angiogenesis, and NAT10 inhibition could induce vascular normalization in gastric cancer.

### VEGFA serves as the principal effector of NAT10-driven angiogenic programming in gastric cancer

**Figure 2:**
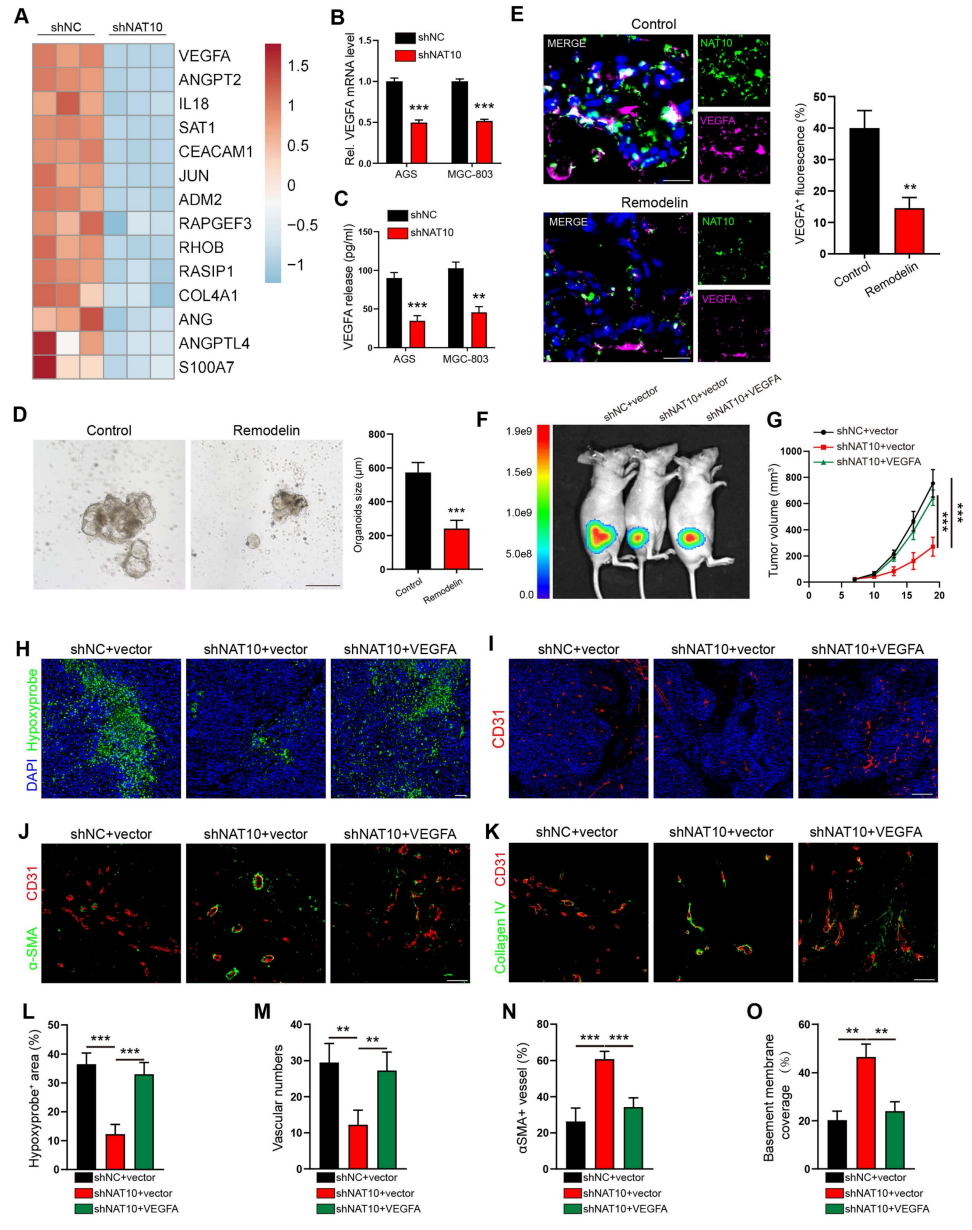
NAT10 enhanced angiogenesis and tumor vessel abnormalization by upregulating VEGFA expression in GC cells. (A) Heatmap depicting significantly downregulated angiogenesis-related genes in NAT10-knockdown cells, as identified by RNA-sequencing. (B) Quantification of VEGFA mRNA levels in shNAT10 cells by qPCR. (C) Measurement of VEGFA protein levels in the culture supernatants of GC cells using enzyme-linked immunosorbent assay (ELISA). (D) Impact of Remodelin on the growth of human-derived gastric tumor organoids. Organoids were quantified, and their sizes were measured. Scale bar: 200 μm. (E) Representative IF images and quantification of VEGFA^+^ cells among 3D organoids. Scale bar: 20 μm. (F, G) Whole-body fluorescence images (F) and growth volume curves (G) of xenograft GC nude mouse model. (H, L) Representative images of Hypoxyprobe-1-labeled areas in MGC803 tumors. Scale bar: 50 μm. (I, M) Blood vessels were stained with the pan-endothelial marker CD31, and the nuclei were stained with DAPI. Scale bar: 200 μm. IF images of αSMA (J, N) and Collagen IV (K, O) in MGC803 tumors. The pericyte coverage and BM coverage were measured using ImageJ software.

Leveraging our prior RNA sequencing dataset, comparative transcriptomic profiling of NAT10-knockdown versus control AGS cells revealed significant downregulation of angiogenesis-related genes^11^. Among 14 differentially expressed angiogenic regulators, we prioritized VEGFA and ANGPT2 based on their established roles in pathological vasculature remodeling (Figure 2A)^18^. However, our qPCR results confirmed that only the VEGFA mRNA level was significantly reduced in both the NAT10-knockdown AGS and MGC-803 cells (Figure 2B and Figure S3A). Furthermore, we detected the protein expression of VEGFA and ANGPT2 in the CM of GC cells by enzyme-linked immunosorbent assay (ELISA). The results showed that knocking down NAT10 significantly reduced the concentration of the VEGFA protein but not the ANGPT2 protein (Figure 2C and Figure S3B), compared to that in the control group. Therefore, we chose VEGFA for further studies and detected its expression by qPCR and IF staining analysis. Consistent with the results of knocking down NAT10 using shRNA, our results showed that inhibiting NAT10 by treatment with Remodelin decreased the expression of VEGFA in human GC cells (Figure S3C, D), mouse tumor cells (MFCs) (Figure S3E, F), and human GC organoid models (Figure 2D, E)^19^.

To verify the role of VEGFA in the vascular development of GC, we knocked down the expression of VEGFA in AGS cells using lentivirus-mediated shRNA (Figure S3I). We found that VEGFA knockdown in GC cells impaired the invasion and tube formation of HUVECs, similar to the observations in NAT10-knockdown cells, which were rescued through lentiviral-mediated ectopic VEGFA expression in GC cells (Figure S3J, K). To further verify the necessity of VEGFA in NAT10-mediated angiogenesis and abnormal tumor vascularization, we transfected NAT10-knockdown GC cells with VEGFA expression plasmids (Figure S3L). As predicted, the invasion and tube formation abilities of HUVECs were reversed after overexpression of VEGFA on the basis of NAT10-silencing (Figure S3M, N). When these cells were injected to generate subcutaneous xenograft GC mouse models, VEGFA re-expression promoted tumor growth, hypoxia and necrosis (Figure 2F-H and L; Figure S3G, H). In addition, our IF staining showed that increased VEGFA expression led to increased vessel density, accompanied by a reduction in αSMA positive pericyte coverage and a decrease in the level of collagen IV in tumor vessels (Figure 2I-K and M-O). These findings suggested that NAT10 enhances angiogenesis and tumor vascular abnormalization by upregulating VEGFA expression in GC cells.

### NAT10 acetylates lncRNA XIST to promote VEGFA expression

**Figure 3:**
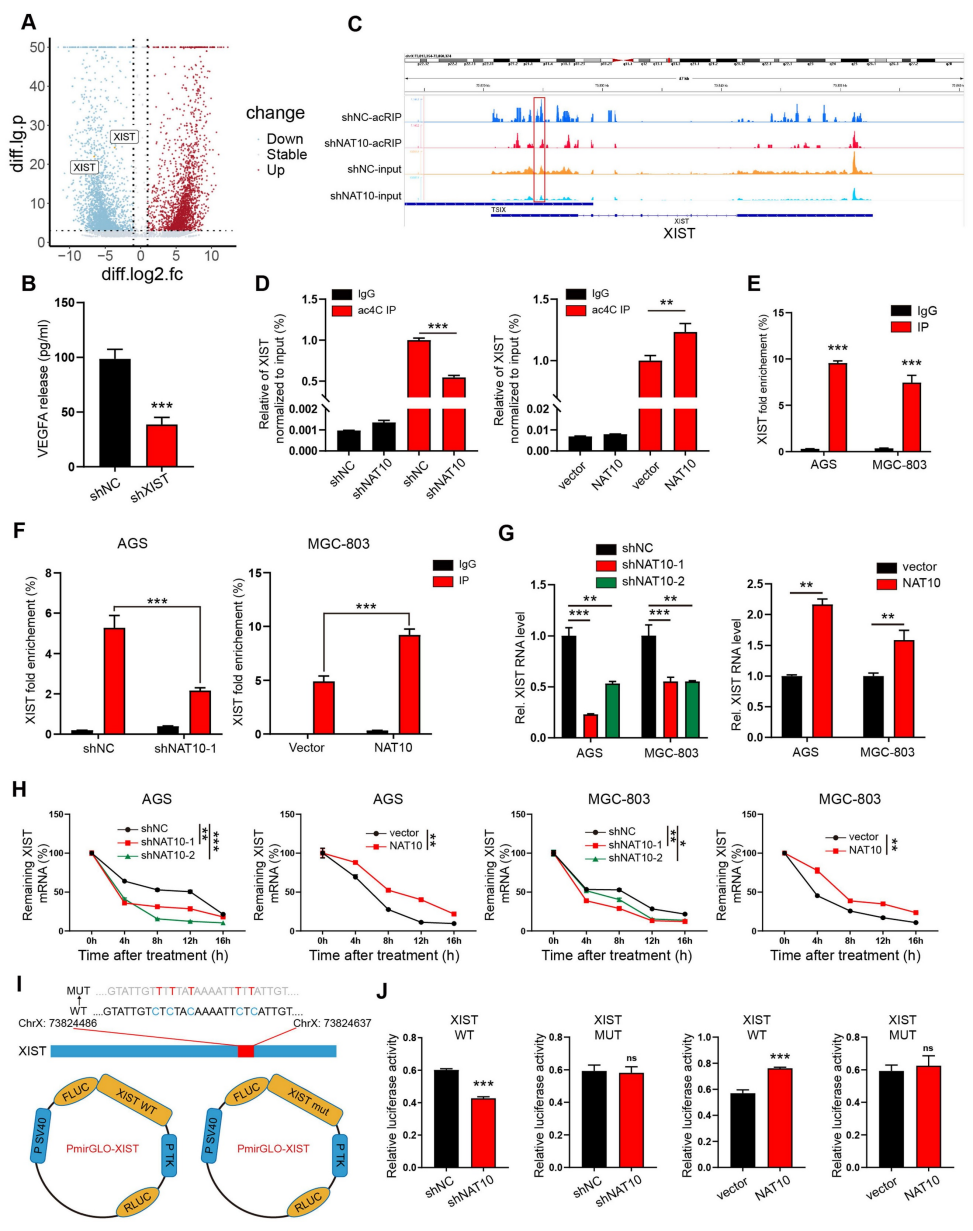
NAT10 acetylates lncRNA XIST to promote VEGFA expression. (A) Volcano plots of differentially acetylated genes identified by acRIP-seq. (B) The level of VEGFA protein in CM collected from shXIST GC cells was measured by ELISA. (C) Distribution of NAT10-binding regions and ac4C peaks on XIST lncRNA visualized by IGV. (D) The acRIP-qPCR assays were performed to detect the ac4C level of XIST in GC cells treated with shNAT10 or NAT10 plasmid. (E) The RIP assays were performed to detect the interaction between NAT10 and XIST in AGS and MGC-803 cells. (F) The interaction between NAT10 and XIST was analyzed by RIP-qPCR in GC cells with NAT10 knockdown or overexpression. (G) The expression level of XIST was detected by qPCR in GC cells treated with shNAT10s or NAT10 plasmid. (H) The mRNA stability was detected by qPCR in AGS and MGC-803 cells treated with actinomycin D (5 μg/ml). (I) Schematic representation of NAT10-targeted ac4C motifs within XIST (upper panel). The ac4C sites within the exon of XIST were mutated and inserted into the pmirGLO plasmid for dual-luciferase assays (lower panel). (J) Dual-luciferase assays were performed in shNAT10 or NAT10-overexpression GC cells using pmirGLO-XIST WT or pmirGLO-XIST mut plasmid.

To elucidate the mechanism underlying NAT10-mediated VEGFA expression in gastric cancer (GC) cells, we re-examined our acetylated RNA immunoprecipitation and sequencing (acRIP-seq) data^11^ . Notably, in VEGFA mRNA, we detected no significant alteration in the ac4C peak-fold between NAT10-knockdown AGS cells and control cells. This finding strongly suggests that NAT10 does not upregulate VEGFA expression through direct acetylation of its mRNA. Subsequently, we aimed to identify potential NAT10 targets that could promote VEGFA expression. Based on the top 20% of differentially acetylated genes (Figure 3A) in the acRIP-seq data, we employed RNA interference (siRNA) and human VEGFA enzyme-linked immunosorbent assay (ELISA) for detection. Notably, knockdown of the long non - coding RNA (lncRNA) XIST in GC cells led to a significant reduction in VEGFA secretion (Figure 3B). Moreover, the elevated VEGFA expression induced by NAT10 overexpression was effectively reversed by XIST knockdown (Figure S4A). Furthermore, we investigated the functional impact of XIST knockdown on endothelial cells. Treatment of human umbilical vein endothelial cells (HUVECs) with conditioned medium (CM) from shXIST GC cells significantly impaired their tube-forming, migratory, and invasive capabilities (Figure S3B, G).

XIST, well-known for its function in the transcriptional silencing of one X-chromosome during the development of female mammals^20^, has recently been reported to play crucial roles in various cancers^21^, ^22^. At our research center, qPCR analysis revealed that XIST expression was significantly elevated in gastric cancer (GC) tissues compared to normal gastric mucosa tissues (Figure S3H). The KM plot (http://kmplot.com) dataset further indicated a negative correlation between XIST expression and the survival of GC patients (Figure S3I). Subsequently, we explored the correlation between ac4C modification and XIST expression. Using IGV, we observed a marked decrease in the enrichment of the ac4C peak of XIST in the shNAT10 immunoprecipitation (IP) group as compared to the control group (Figure 3C). Consistent with the acRIP-seq results, acRIP followed by qPCR verified a substantial reduction in the ac4C abundance of XIST in NAT10-knockdown AGS cells. Conversely, NAT10 overexpression led to an increase in ac4C abundance of XIST (Figure 3D). RNA-immunoprecipitation (RIP) assays clearly demonstrated an interaction between NAT10 and XIST (Figure 3E). Moreover, the enrichment of XIST associated with NAT10 was altered upon NAT10 knockdown or overexpression (Figure 3F). Additionally, qPCR data showed that NAT10 knockdown decreased XIST expression, while NAT10 overexpression increased it (Figure 3G). As previously reported, NAT10 could modulate gene expression by regulating RNA stability or translation efficiency^8^, ^23^. To investigate the effect of NAT10 on XIST stability, we treated AGS and MGC-803 cells with actinomycin D to assess RNA decay following NAT10 knockdown or overexpression. As anticipated, XIST degraded at a much faster rate in NAT10-knockdown cells compared to control cells, and NAT10 overexpression reversed this effect (Figure 3H). We furtherly constructed luciferase reporter plasmids encoding either wild-type XIST (XIST-WT) or a mutant form (XIST-MUT) (Figure 3I). In the XIST mutant, cytosine (C) was substituted with thymine (T) at the ac4C peak. The dual-luciferase assay confirmed that NAT10 regulated the stability of XIST, and this regulation was contingent upon the ac4C modification of XIST lncRNA exons (Figure 3J). Collectively, these results suggest that NAT10 promotes VEGFA expression and angiogenesis in gastric cancer by acetylating the lncRNA XIST.

### NAT10/XIST promotes VEGFA expression by facilitating nuclear accumulation of the YAP1 protein

**Figure 4:**
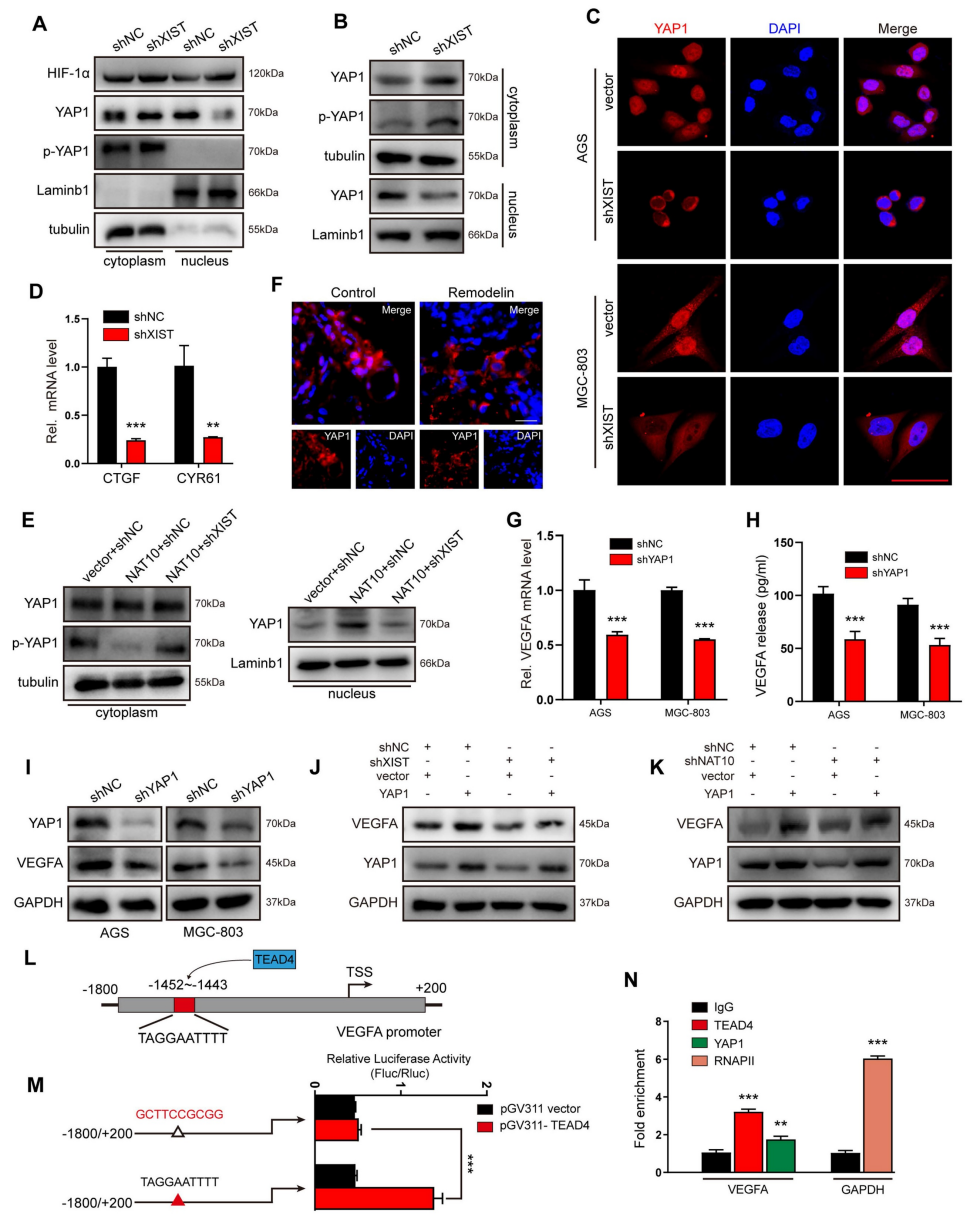
NAT10/XIST facilitates VEGFA expression by promoting the nuclear transport of YAP1. (A) The protein expression of HIF-1α and YAP1 in nuclear and cytoplasmic component in shXIST-knockdown AGS cells (treated with 100 μM CoCl2). (B) A nucleus-cytoplasm separation assay was performed to detect the expression of YAP1 in AGS cells under normoxic conditions. (C) The location of YAP1 was detected by IF staining in shXIST GC cells. Scale bar: 100 μm. (D) The mRNA levels of CTGF and CYR61 were detected in shXIST cells by qPCR. (E) A nucleus-cytoplasm separation assay was performed to detect YAP1 expression in the nuclear and cytoplasmic components of AGS cells. (F) Representative images showing YAP1 expression (red) in Remodelin-treated GC organoids. Scale bar: 20 μm. (G-I) The mRNA and protein levels of VEGFA in shYAP1-GC cells were analyzed by qPCR (G) and western blot (I); The VEGFA protein in CM was detected by ELISA(H). (J, K) The expression of VEGFA and YAP1 proteins in shXIST- or shNAT10-AGS cells transfected with a YAP1 plasmid was detected by western blot. (L) Illustration of TEAD4 protein targeting to VEGFA promoter. (M) Dual-luciferase reporter assay was performed to confirm TEAD4 targeted to the promoter of VEGFA gene. (N) ChIP-qPCR analysis of TEAD4 binding to the VEGFA promoter region in MGC-803 and AGS cells. RNA polymerase II (RNAPII) was used as a positive control. The fold enrichment over the IgG control is represented.

Hypoxia-inducible factor-1α (HIF-1α) is pivotal in angiogenesis, as it activates the transcription of angiogenesis-associated genes, including VEGFA. Our team previously reported that NAT10 promotes the translocation of HIF-1α into the nucleus by acetylating SEPT9 mRNA^11^, ^24^. To determine whether XIST also promotes VEGFA expression by facilitating HIF-1α nuclear translocation, we examined the presence of HIF-1α protein in the nucleus of XIST-knockdown cells. Under cobalt chloride (CoCl₂)-induced hypoxic conditions, the levels of HIF-1α protein in the nucleus of shXIST AGS cells showed no significant change (Figure 4A). This finding indicates that the NAT10/XIST axis does not promote VEGFA expression by enhancing the nuclear accumulation of HIF-1α protein. Unexpectedly, we observed that the expression of Yes-associated protein 1 (YAP1), another well-recognized transcription factor related to angiogenesis^25^, was significantly reduced in the nuclei of shXIST AGS cells under both hypoxic and normoxic conditions (Figure 4A-C). Moreover, the phosphorylation of the YAP1 protein at Ser127 (YAP1-Ser127), which prevents its nuclear translocation^26^, was significantly increased in shXIST AGS cells (Figure 4A, B). Reintroduction of XIST effectively decreased the total amount of YAP1-Ser127 protein and promoted YAP1 nuclear translocation in shXIST AGS cells (Figure S4A). We also found that XIST knockdown significantly decreased the expression of YAP1 target genes CYR61 and CTGF (Figure 4D), suggesting that XIST knockdown inhibits the transcriptional activity of YAP1. Overexpression of NAT10 significantly upregulated YAP1 nuclear translocation, an effect that could be reversed by knocking down XIST (Figure 4E and Figure S5B). Additionally, inhibition of NAT10 function in GC organoids using Remodelin led to a significant reduction in nuclear YAP1 levels (Figure 4F). Finally, overexpression of YAP1 in either shNAT10 or shXIST GC cells significantly reversed the reduction in angiogenesis in chicken embryos and the decline in tube formation, migration, and invasion in human umbilical vein endothelial cells (HUVECs) (Figure S5C-F). These results suggest that the NAT10/XIST axis promotes the nuclear accumulation of YAP1 and angiogenesis in gastric cancer.

YAP1, a potent transcriptional co-activator within the Hippo pathway, exerts its function by binding to TEAD transcription factors (TEAD1-4) and plays a pivotal role in the progression of gastric cancer^27^, ^28^. To verify whether YAP1 regulates the transcription of VEGFA, we silenced YAP1 in GC (AGS and MGC-803) cells and subsequently examined VEGFA expression. The results demonstrated that VEGFA expression was positively correlated with YAP1 expression at both the mRNA and protein levels (Figure 4G-I). Furthermore, rescue experiments revealed that YAP1 overexpression effectively counteracted the reduction in VEGFA expression induced by either XIST or NAT10 knockdown (Figure 4J, K). To identify which TEAD transcription factor predominantly promoted YAP1-mediated VEGFA expression, we transfected AGS cells with TEAD1-4 siRNAs. qPCR results indicated that TEAD4 most significantly inhibited VEGFA expression in GC cells (Figure S4G). Subsequently, potential binding sites for TEAD4 in the VEGFA promoter region were predicted using the JASPAR website (Figure 4L). To confirm that TEAD4 binds to the VEGFA promoter to promote its transcription, dual-luciferase reporter and chromatin immunoprecipitation (ChIP) assays were performed. The luciferase reporter assay showed a significant decrease in reporter activity in AGS cells co- transfected with pGV311-TEAD4 and pGL3-VEGFA-903-mut (Figure 4M) compared to those co- transfected with pGV311-TEAD4 and pGL3-VEGFA-903. ChIP-qPCR analysis revealed that VEGFA promoter sequences were specifically enriched by anti-YAP1 and anti-TEAD4 antibodies, but not by the negative control antibody IgG in AGS cells (Figure 4N). Collectively, these data suggest that the YAP1/TEAD4 complex promotes the transcription of the VEGFA gene in GC cells.

### XIST recruits hnRNPK to promote nuclear translocation and activation of the YAP1 protein

**Figure 5:**
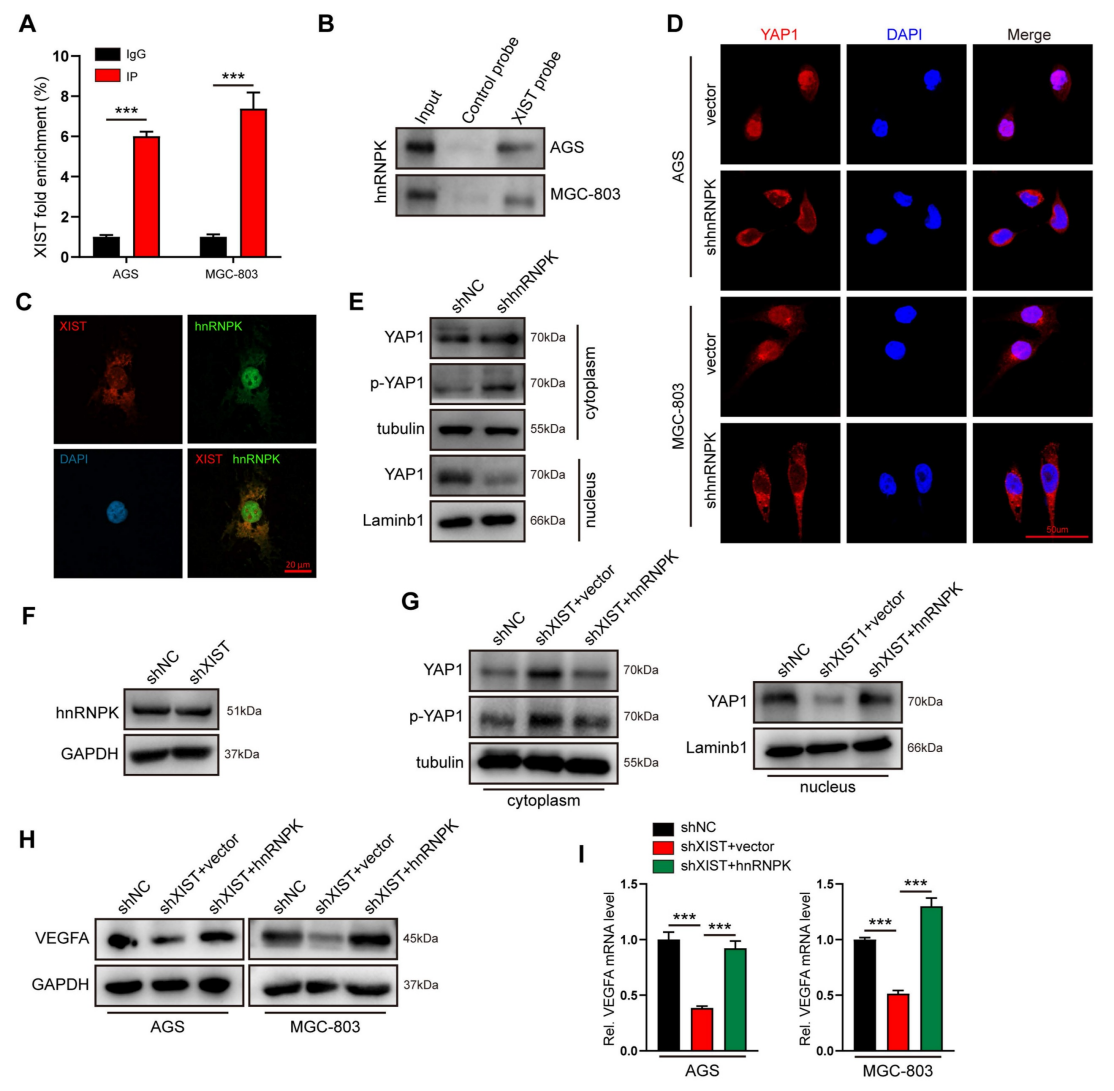
XIST recruits hnRNPK to facilitate YAP1 nuclear translocation. (A) hnRNPK RIP followed by qPCR for XIST expression in AGS and MGC-803 cells. (B) RNA pull-down was performed to investigate the interaction between hnRNPK and XIST, biotin-labeled XIST was incubated with GC cell lysates, and the enriched hnRNPK was detected by western blot. (C) The co-localization of XIST and hnRNPK in AGS was detected by FISH-IF assay. (D) The location of YAP1 was detected by IF staining in shhnRNPK-GC cells. Scale bar: 50 μm. (E) A nucleus-cytoplasm separation assay was performed to detect YAP1 and p-YAP1 expression in the nuclear and cytoplasmic components of shhnRNPK-AGS cells. (F) Western blot for hnRNPK expression in shXIST-GC cells. (G) A nucleus-cytoplasm separation assay was performed to detect the expression of YAP1 in AGS cells. (H, I) the protein and mRNA level of VEGFA were detected by qPCR and western blot.

How does XIST facilitate the nuclear accumulation of YAP1 proteins? The comprehensive identification of RNA-binding proteins by mass spectrometry (ChIRP-MS) has enabled the systematic discovery of XIST RNA-binding proteins in various cell types. Heterogeneous nuclear ribonuclear protein K (hnRNPK), which has been recently reported to collaborate with YAP to enhance gene transcription^29^, has been confirmed as an XIST RNA-binding protein in chromatin modifications^30^. Thus, we hypothesized that XIST recruits hnRNPK to promote the nuclear translocation of the YAP1 protein and YAP1/TEAD4-mediated VEGFA transcription in gastric cancer cells. To test this hypothesis, we initially verified the physical interaction between XIST and hnRNPK in AGS cells using RNA-immunoprecipitation (RIP), RNA pull-down assays, and fluorescence in situ hybridization (FISH) (Figure 5A-C). Subsequently, immunofluorescence (IF) and nuclear-cytosol fractionation assays demonstrated that the nuclear localization of YAP1 was significantly inhibited in hnRNPK-knockdown GC cells. This finding suggests that hnRNPK also functions as a YAP1-binding partner in GC cells (Figure 5D, E). Furthermore, we observed that overexpression of hnRNPK restored the reduced nuclear accumulation of YAP1 and the expression of VEGFA that were caused by XIST silencing (Figure 5G-I). Notably, knocking down XIST expression had no impact on hnRNPK expression (Figure 5F), indicating that hnRNPK is essential for XIST-mediated YAP1 nuclear translocation and VEGFA expression. In summary, XIST promotes the nuclear translocation and activation of YAP1 in an hnRNPK - dependent manner.

### NAT10 inhibition promotes immune reprogramming and sensitizes mouse tumors to anti-PD-1 immune checkpoint therapy

**Figure 6:**
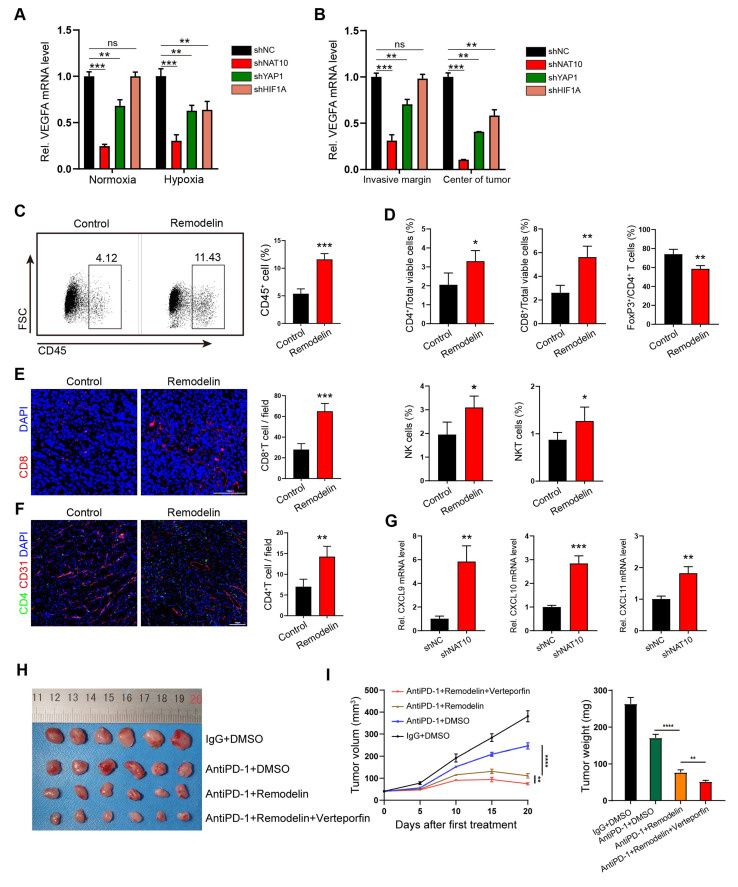
The NAT10 inhibitor Remodelin promotes immune reprogramming and sensitizes GC tumors to anti-PD-1 therapy. (A, B) The mRNA levels of VEGFA in shNAT10, shYAP1 and shHIF1A-GC cells/tumors were detected by qPCR. (C, D) The proportions of CD45+ leukocytes (C) and the densities of different immune cells (D) were determined by flow cytometry. (E, F) Representative images and quantification of CD8+ T cells and CD4+ T cells within subcutaneous MFC tumors treated with Remodelin. Scale bar: 100μm. (G) qPCR analysis of CXCL9, CXCL10 and CXCL11 expression in shNAT10-GC cells. (H-I) Representative images (H) of MFC tumors, tumor growth curves and tumor weights (I) are shown; Data were shown as mean ± SEM.

Building on the preceding findings, we have demonstrated that the NAT10/XIST/YAP1 axis in GC cells drives the upregulation of VEGFA and leads to vascular abnormalization. Given our previous reports that the NAT10/SEPT9/HIF-1α positive feedback loop governs glycolysis addiction and that HIF-1α can also foster VEGFA-associated angiogenesis within the hypoxic tumor microenvironment^11^, ^31^, we detected the expression of VEGFA mRNA in three groups of gene deficient GC cell lines (shNAT10, shYAP1 and shHIF1A GC cells) under both normoxic and hypoxic (100 µM CoCl2) conditions by qPCR. As expected, the VEGFA mRNA levels in shNAT10-GC cells were lower than those in shYAP1- or shHIF1A-GC cells under both normoxic and hypoxic conditions (Figure 6A), which suggested that compared with intervening in downstream YAP1 or HIF, inhibiting NAT10 can downregulate VEGFA expression more significantly. We also found that the VEGFA mRNA level in normoxic shHIF1A-GC cells was not significantly different from that in the corresponding negative control (normoxic shNC-GC cells) (Figure 6A), supporting that HIF-1α is degraded in cells under normoxic conditions^32^. In contrast, the VEGFA mRNA level in both normoxic and hypoxic shYAP1 GC cells was significantly lower than that in negative control cells (Figure 6A), albeit this reduction was less pronounced than that in shNAT10 GC cells. This indicates that targeting NAT10 in GC cells has the most substantial impact on inhibiting VEGFA expression. When these gene-deficient GC cells (shNAT10-, shYAP1-, and shHIF1A- GC cells) were subcutaneously transplanted into nude mice, we examined the expression of VEGFA mRNA and the structure of the tumor vascular network. The results further indicated that knocking down NAT10 most effectively inhibited VEGFA expression, angiogenesis, and vascular abnormalization (Figure 6B and Figure S6).

To determine whether targeting NAT10-induced vascular normalization could effectively promote immune reprogramming within the tumor microenvironment, we employed flow cytometry analysis and immunofluorescence (IF) staining to analyze the infiltrating immune cell populations in tumors treated with Remodelin. The findings demonstrated that the density of CD45+ cells was significantly higher in Remodelin - treated tumors compared to control tumors (Figure 6C). This suggests that the normalization of vascular fluid pressure facilitates immune cell infiltration. Furthermore, we observed increased infiltration of CD4+ T, CD8+ T, natural killer (NK), and natural killer T (NKT) cells in Remodelin-treated tumors (Figure 6D-F). Conversely, the infiltration of regulatory T (Treg) cells was reduced in these tumors (Figure 6D). Consistent with these results, our transcriptome data indicated that the expression levels of inflammatory CXC chemokines (CXCL9, CXCL10, and CXCL11) were significantly upregulated in shNAT10-GC cells (Figure S7A), which was further validated by qPCR analysis (Figure 6G). The negative correlation between NAT10 and CXCL9/10/11 was further confirmed in Remodelin-treated or NAT10-overexpressing GC cells (Figure S7B, C). Given that CXCL9/10/11 have been reported to recruit cytotoxic lymphocytes (CTLs), natural killer cells (NKs), and NKT cells^33^, ^34^, they may act in concert with VEGFA to promote NAT10-induced vascular normalization and immune reprogramming.

**Figure 7:**
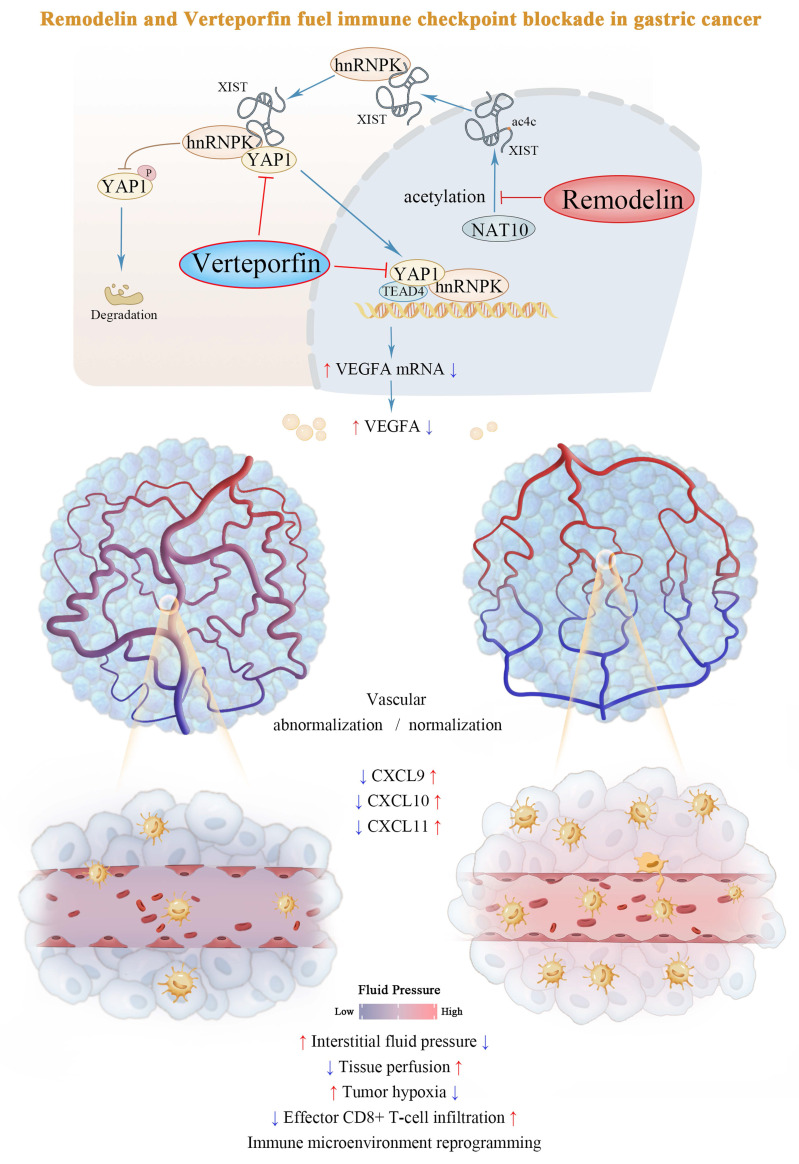
Schematic model of the NAT10/XIST/YAP1 axis in GC.

To further explore whether targeting the NAT10/XIST/YAP1 axis augments the antitumor effect of immune checkpoint inhibitors (ICIs), we administered Remodelin, Verteporfin, and an anti-PD-1 monoclonal antibody to tumor-bearing mice. The results revealed that the NAT10 inhibitor Remodelin significantly enhanced the efficacy of PD-1 therapy. Notably, when combined with the YAP1 inhibitor Verteporfin, an even more pronounced therapeutic effect was achieved (Figure 6H, I). Overall, our results suggest that Remodelin and Verteporfin can potentiate immune checkpoint blockade in gastric cancer (Figure 7).

## Discussion

Mounting evidence positions vascular normalization as a critical determinant of immune checkpoint inhibitor efficacy^1^. Our study elucidates a previously unrecognized epigenetic circuit wherein the NAT10/XIST/YAP1 axis promoted VEGFA transcription and vascular abnormalization under both normoxic and hypoxic conditions. We further demonstrated that the NAT10 inhibitor, Remodelin, effectively reversed vascular abnormalities, facilitated immune reprogramming in the TME. Moreover, it synergistically interacted with the YAP1 inhibitor, Verteporfin, to render mouse tumors more sensitive to anti-PD-1 immune checkpoint therapy (Figure 7). Taken together, targeting the NAT10/XIST/YAP1 axis-mediated vascular abnormalization by using Remodelin and Verteporfin could enhance immune checkpoint blockade in gastric cancer.

Emerging evidence establishes NAT10, the principal RNA acetyltransferase catalyzing N4-acetylcytidine (ac4C) modifications, as a master coordinator of post-transcriptional regulation through enhanced mRNA stability and translational fidelity^12^-^15^. Building upon our prior discovery of the NAT10/SEPT9/HIF-1α positive feedback loop driving glycolytic addiction in GC^11^, this work unveils a novel NAT10/XIST/YAP1/VEGFA axis governing tumor vascular ecology. Since our transcriptome data were collected from GC cells under normoxic culture conditions and HIF-1α was constantly synthesized but rapidly degraded under normoxic conditions^35^, the NAT10/XIST/YAP1 axis might play a more important role in the entire cycle of tumor formation, particularly during the early tumorigenesis phase. Moreover, we confirmed Remodelin could targeting the NAT10/YAP1/VEGFA axis to enhance vascular fluid pressure and promote immune cell infiltration, and found the combination of Remodelin and Verteporfin enhances the efficacy of anti-PD-1 immune checkpoint therapy in 615 mouse allografts. Our study establishes a novel combination therapeutic strategy to potentiate immune checkpoint blockade efficacy in gastric cancer, providing a clinically translatable approach to enhance anti-PD-1 responsiveness through vascular-immune microenvironment remodeling.

In this study, we identified the lncRNA XIST as a direct target for NAT10-mediated ac4c modification in GC cells, providing support for the presence of ac4c modification in noncoding RNAs^36^. Although XIST was initially recognized as a key regulatory factor of X-chromosome inactivation in female cells^37^. recent studies have shown that XIST is somatically activated in male human cancers and acts as a novel oncogene in multiple cancers, including GC^22^, ^36^, ^38^. Our study highlighted NAT10-mediated ac4c modification as a key mechanism driving the high expression of XIST in GC cells. We discovered a new mechanism by which XIST promotes vascular abnormalization by recruiting hnRNPK and enhancing the activation of the YAP1 protein in both female and male GC cell lines^29^.

Looking ahead, the existence of ac4c modifications in other noncoding RNAs, such as circRNAs, and their biological functions, remain to be elucidated. Although we have established that the inhibition of NAT10 significantly upregulates the inflammatory chemokines (CXCL9, CXCL10, and CXCL11) to promote immune reprogramming, the precise underlying mechanism remains to be further investigated.

## Conclusions

In summary, our study uncovered that NAT10-mediated ac4C modification stabilizes lncRNA XIST, which recruits hnRNPK to facilitate YAP1 nuclear translocation and transcriptional activation, thereby driving VEGFA-dependent vascular abnormalization in gastric cancer. The NAT10 inhibitor Remodelin could synergize with the YAP1 inhibitor Verteporfin to fuel immune checkpoint blockade in gastric cancer.

## Supplementary Material

Supplementary figures and tables.

## Abbreviations

ICI: immune checkpoint inhibitor
TME: tumor microenvironment
GC: gastric cancer
ac4C: N4-acetylcytidine
DAPI: 4',6-diamidino-2-phenylindole
CT: center of tumor
IM: invasive margin
α-SMA: α-smooth muscle actin
BM: basement membrane
CM: conditioned medium
CAM: chick embryo chorioallantoic membrane
HIF-1α: hypoxia-inducible factor-1α
CoCl2: cobalt chloride
