# Supplementary material for: Targeting the NAT10/XIST/YAP1 Axis-Mediated Vascular Abnormalization Enhances Immune Checkpoint Blockade in Gastric Cancer
Source: Int J Biol Sci. 2025 Jul 28;21(11):4997–5014. doi: 10.7150/ijbs.113325 (PMC12374829; doi:10.7150/ijbs.113325)
Supplement: Supplementary file 1 — Supplementary figures and tables. [file ijbsv21p4997s1.pdf]

# Supplementary Information for

## *Targeting the NAT10/XIST/YAP1 Axis-Mediated Vascular Abnormalization Enhances Immune Checkpoint Blockade in Gastric Cancer*

Xuetao Lei *et al.*

### Supplementary data

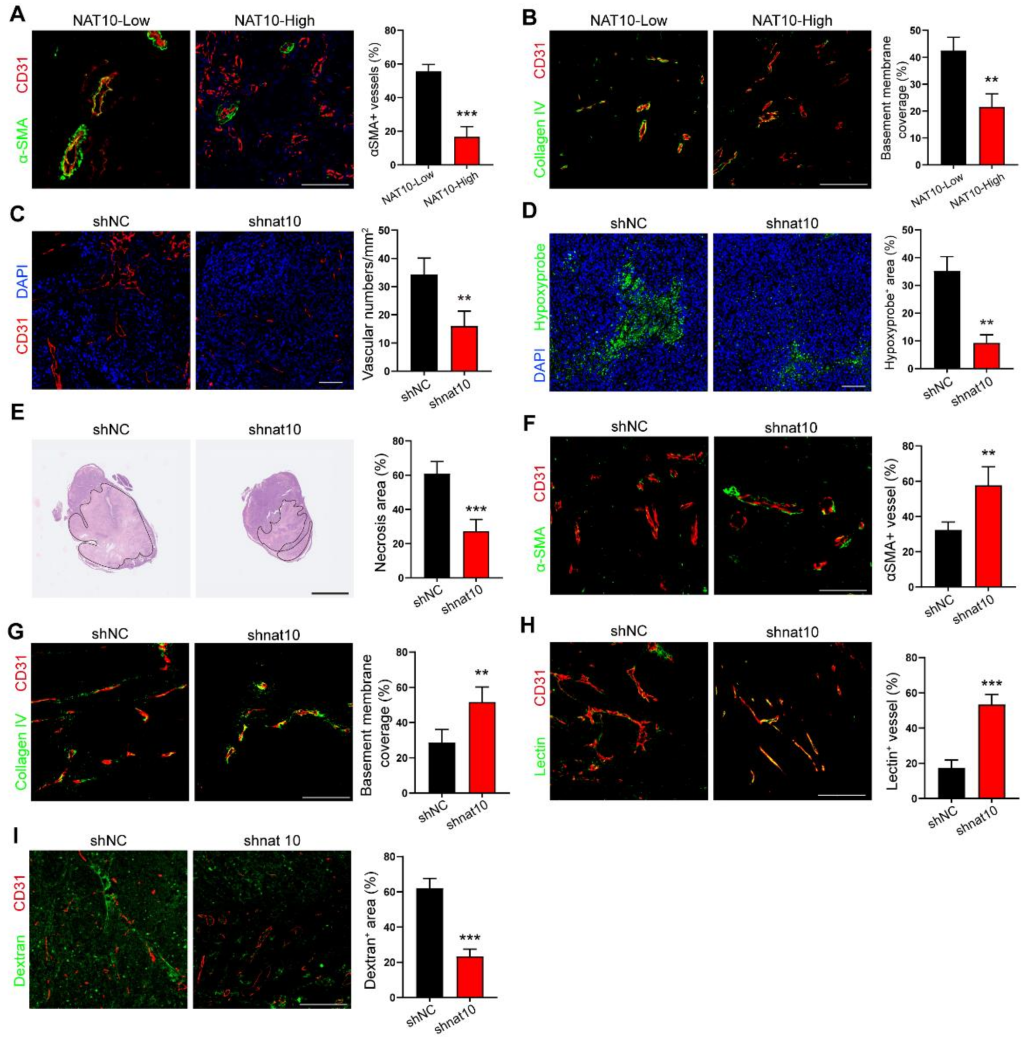


Figure S1. NAT10 knockdown in gastric cancer (GC) cells suppresses tumor angiogenesis and induces vascular normalization in vivo.

(A, B) Immunofluorescence (IF) staining of αSMA (pericytes, A) and collagen IV (basement membrane, B) in human GC tumors stratified by NAT10 expression

(NAT10-low vs. NAT10-high groups). Quantitative analysis of pericyte coverage and basement membrane (BM) coverage was performed using ImageJ (mean ± SEM, n=10 tumors/group). Scale bars: 50 µm. (C) $CD31^{+}$ vessel density in shnat10-MFC tumors compared to controls. Scale bar: 50 µm. (D) Representative images of Hypoxyprobe-1-labeled areas in shnat10-MFC tumors. Scale bar: 50 µm. (E) Representative images of shnat10-MFC tumor necrosis. The H&E staining was conducted and the necrosis area was labeled using lines. Scale bar: 2.5 mm. (F, G) IF staining of αSMA (F) and collagen IV (G) in shnat10-MFC tumors. Pericyte and BM coverage were quantified (mean ± SEM). Scale bars: 50 µm. (H, I) Representative images for vessels perfused with lectin (H) and dextran (I) in shnat10-MFC tumors. Scale bar: 50 µm.

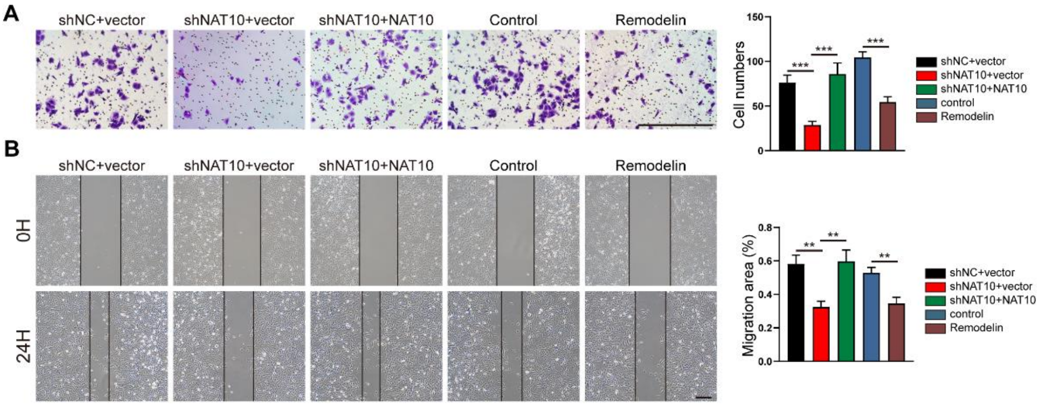


Figure S2. NAT10 enhances tube formation and migration of HUVECs.

(A) Transwell invasion and (B) wound healing migration assays of HUVECs incubated with conditioned medium (CM) from AGS cells subjected to NAT10 knockdown (shRNA) or pharmacological inhibition Remodelin. Three independent experiments were performed. Scale bar: 500 µm.

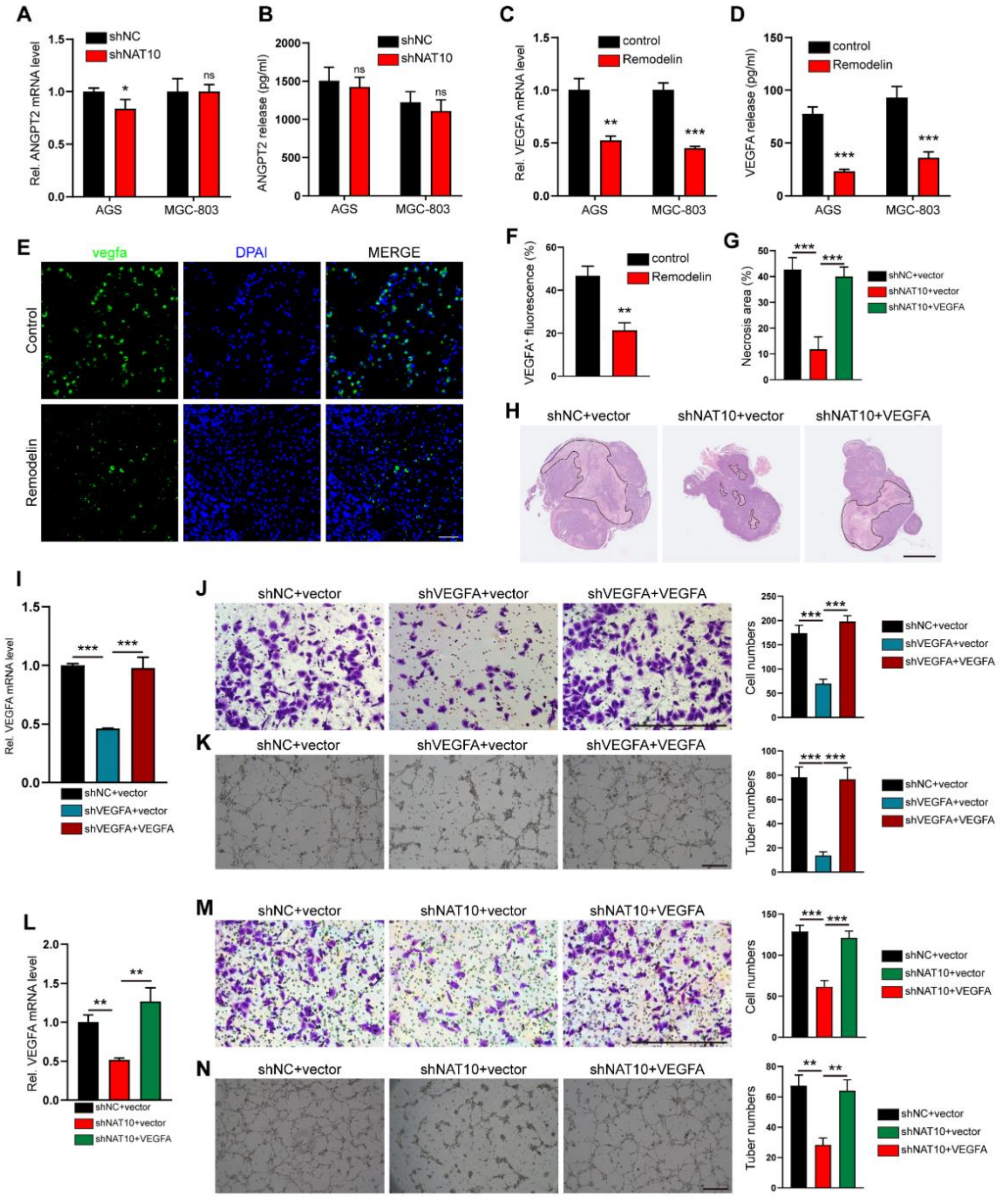


Figure S3. VEGFA is required for NAT10-mediated angiogenesis. (A, B) The mRNA level of ANGPT2 in shNAT10 GC cells were analyzed by qPCR (A), and the ANGPT2 protein level in CM were detected by ELISA (B). (C, D) The mRNA level of VEGFA in Remodelin-treated GC cells were analyzed by qPCR (C), and the VEGFA protein levels in CM were detected by ELISA (D). (E, F) Representative IF images (E) and quantification of VEGFA+ cells (F) in MFC tumors. scale bars, 50 μm. (G, H) Representative images of GC tumor necrosis. The H&E staining was conducted and necrosis area was labeled by lines. Scale bar, 2.5 mm. (I-K) qPCR for VEGFA expression in VEGFA-knockdown AGS cells with VEGFA re-expression (I), and the invasion abilities (J) and impaired tuber formation (K) of HUVECs were shown. Scale bar, 500 μm. (L-N) Rescue of VEGFA expression in NAT10-knockdown AGS cells via VEGFA overexpression (qPCR, L), and functional validation showing HUVEC invasion (M) and tube formation (N) following exposure to CM from these AGS cells. Scale bar: 500 μm.

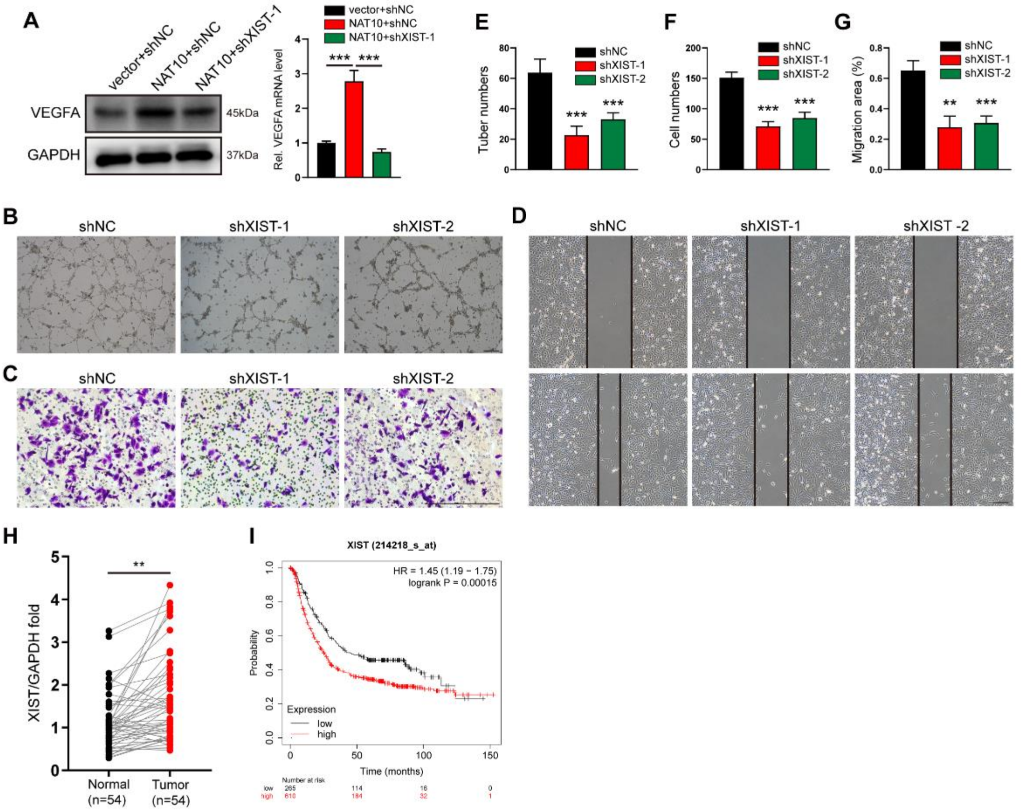


Figure S4. NAT10 mediates VEGFA upregulation and gastric cancer (GC) angiogenesis via lncRNA XIST acetylation. (A) Western blot and qPCR analysis of VEGFA expression in NAT10-knockdown AGS cells transfected with shXIST-1. (B-G) The abilities of tube formation (B, E), migration (C, F) and invasion (D, G) in HUVECs were detected. Scale bar, 500 μm. (H) qPCR analysis of XIST expression in paired GC and adjacent normal tissues (n=54). (I) Kaplan-Meier analysis using an independent cohort (KM Plotter database, http://kmplot.com) showing negative correlation between high XIST expression and overall survival in GC patients.

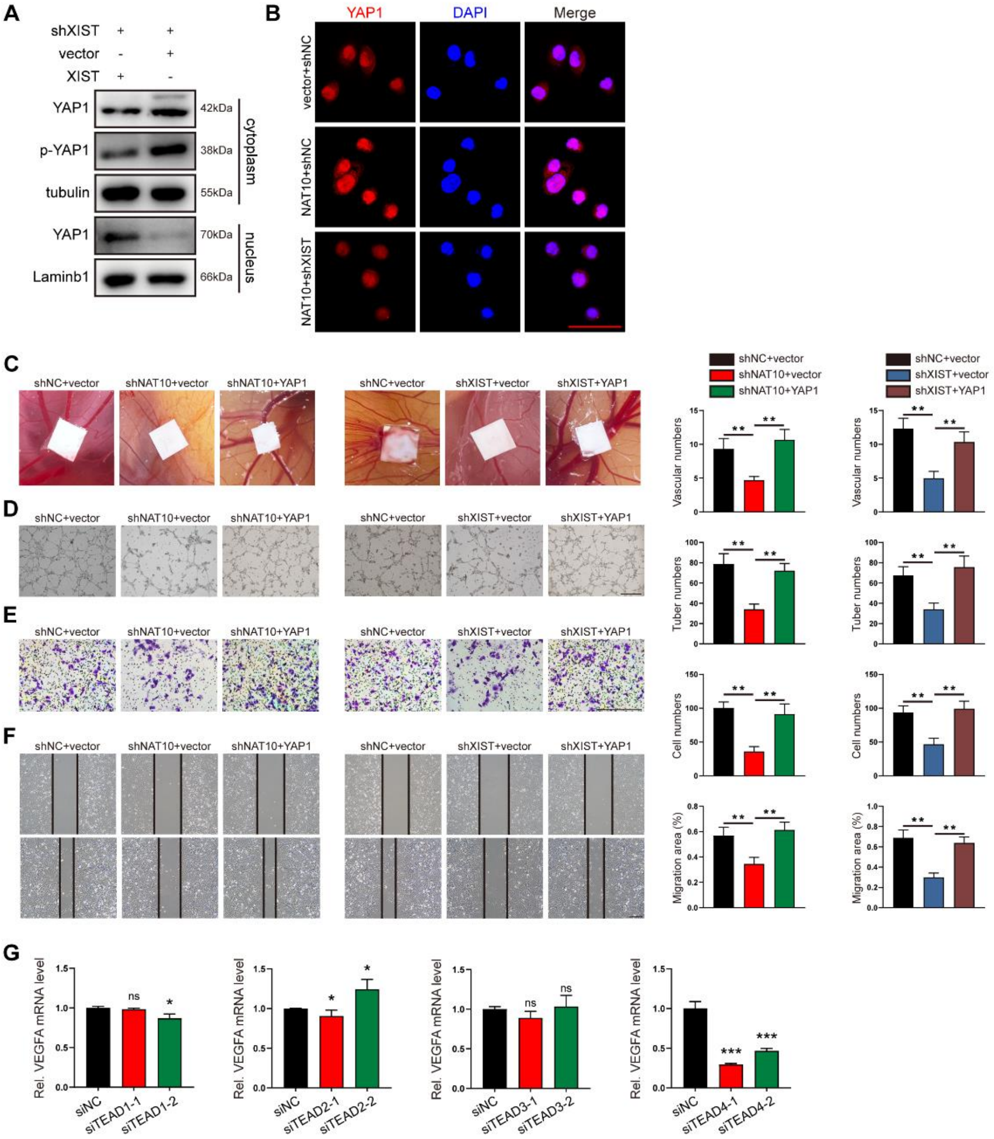


Figure S5. YAP1 serves as a critical downstream mediator of the NAT10/XIST axis in angiogenesis. (A) Subcellular fractionation assay (western blot) showing YAP1 and phosphorylated YAP1 (p-YAP1) expression in AGS cells. (B) Immunofluorescence analysis of YAP1 nuclear translocation in NAT10-overexpressing AGS cells with XIST knockdown. Scale bar: 50 μm. (C) Conditioned medium (CM) from YAP1-overexpressing AGS cells rescued the inhibitory effects of shNAT10 or shXIST on angiogenesis in the chorioallantoic membrane (CAM) assay. (D-F) CM from YAP1-overexpressing AGS cells reversed the suppression of HUVEC tube formation (D), Transwell migration (E), and Matrigel invasion (F) induced by shNAT10 or shXIST. Scale bar: 500 μm. (G) qPCR analysis of VEGFA expression in AGS cells following siRNA-mediated knockdown of TEAD1-4.

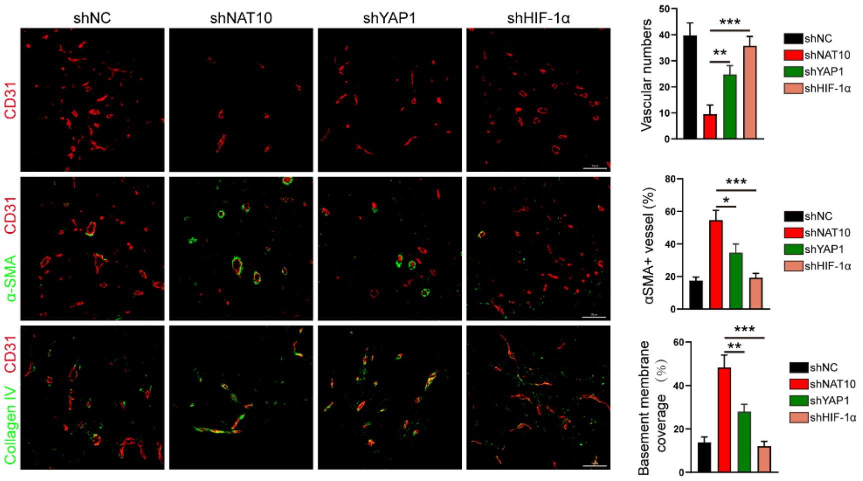


Figure S6. Immunofluorescence analysis of CD31, αSMA, and collagen IV in murine tumor tissues. Vascular parameters, including vessel density, pericyte coverage, and basement membrane integrity, were quantified using ImageJ (NIH). Data represent mean ± SEM from three independent experiments. Scale bar: 50 μm.

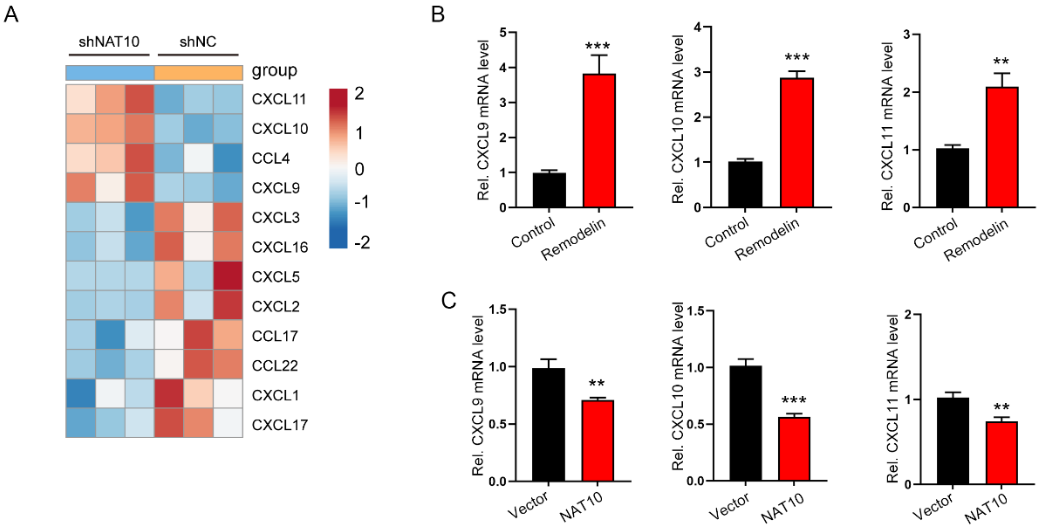


Figure S7. Inhibition of NAT10 enhances the expression of chemokines CXCL9, CXCL10, and CXCL11. (A) Heatmap of differential CXC chemokine expression in NAT10-knockdown versus control cells (RNA-seq). (B) qPCR analysis of CXCL9, CXCL10, and CXCL11 expression in AGS cells treated with the NAT10 inhibitor Remodelin. (C) qPCR analysis of CXCL9, CXCL10, and CXCL11 expression in NAT10-overexpressing AGS cells.

**Table S1. Summary of plasmids used in this study.**

| Plasmids | | Catalog number | Source |
|---|---|---|---|
| shRNA plasmids | shNAT10 | HSH062617-LVRU6GP | GeneCopoeia |
| | shXIST | CS-SH3643-LVRU6GH | |
| | shhnRNPK | HSH100318-LVRU6GP | |
| | shYAP1 | HSH063250-LVRU6GP | |
| | shVEGFA | HSH018480-LVRU6GP | |
| | shNC | CSHCTR001-LVRU6GP | |
| | shNat10 | MSH039885-LVRU6GP | |
| Overexpression (OE) plasmid | Vector | EX-NEG-Lv201 | GeneCopoeia |
| | NAT10 | EX-I5674-Lv201 | |
| | VEGFA | EX-Z2831-Lv242 | |
| | hnRNPK | EX-A6972-Lv102 | |
| | YAP1 | EX-A4844-Lv130 | |
| siRNA | TEAD1 | siG000007003 | Ribobio |
| | TEAD2 | siG000008463 | |
| | TEAD3 | siG000007005 | |
| | TEAD4 | siG000007004 | |

**Table S2. The primer sequences used for qPCR in this study**

| primers | 5' to 3' |
|---|---|
| NAT10-For | ATAGCAGCCACAAACATTCGC |
| NAT10-Rev | ACACACATGCCGAAGGTATTG |
| GAPDH-For | AAGGTCGGAGTCAACGGA |
| GAPDH-Rev | TTAAAAGCAGCCCTGGTGA |
| YAP1-For | GGTTTCCCTGCTTTCCA |
| YAP1-Rev | CTGCCCCAACCAGATTTA |
| XIST-For | GCAACAACCCTAGGTCAGGA |
| XIST-Rev | AGCTCTCTGCACTGCTTGTAG |
| VEGFA-For | AGGGCAGAATCATCACGAAGT |
| VEGFA-Rev | AGGGTCTCGATTGGATGGCA |
| ANGPT2-For | AACTTTCGGAAGAGCATGGAC |
| ANGPT2-Rev | CGAGTCATCGTATTCGAGCGG |
| hnRNPK -For | CAATGGTGAATTTGGTAAACGCC |
| hnRNPK -Rev | GTAGTCTGTACGGAGAGCCTTA |
| CXCL9 -For | TGGTAAAACACTTGCGGAT |
| CXCL9 -Rev | TTGTATGGTTGGGAAAAGGT |
| CXCL10 -For | GTGGCATTCAAGGAGTACCTC |
| CXCL10 -Rev | TGATGGCCTTCGATTCTGGATT |
| CXCL11 -For | GACGCTGTCTTTGCATAGGC |
| CXCL11 -Rev | GGATTTAGGCATCGTTGTCCTTT |
| Nat10 -For | TCATTGAGAATGGCGTAGCTG |
| Nat10 -Rev | CACAGTTGCCTTGGACAACAT |

**Table S3. The antibodies used in this study**

| Primary antibodies | Catalog number | Source |
|---|---|---|
| Anti-NAT10 (1:1000) | 13365-1-AP | proteintech |
| Anti-N4-acetylcytidine(ac4C) (1:1000) | ab252215 | Abcam |
| Anti-GAPDH (1:1000) | 10494-1-AP | proteintech |
| Anti-YAP1 (1:200) | 13584-1-AP | proteintech |
| Anti-HIF-1α (1:500) | H1alpha67 | Novus |
| Anti-hnRNPK(1:1000) | 11426-1-AP | proteintech |
| Anti-CD31 (1: 500) | ab182981 | Abcam |
| Anti- collagen IV (1: 100) | ab23640 | Abcam |
| Anti-αSMA (1: 100) | AF1032 | Affinity |
| Anti- CD8 (1: 200) | ZA-0508 | ZSGB-BIO |
| Anti- CD4 (1: 500) | RMA-0620 | MXB |
| Anti- VEGFA (1: 200) | 19003-1-AP | Proteintech |
| DAPI | #4083 MSDS | Cell Signaling Technology |
| Anti-Mouse IgG H&L (HRP) | Ab136815 | Abcam |
| Anti-Rabbit IgG H&L (HRP) | Ab136817 | Abcam |
| Anti-Rabbit IgG (H+L), F(ab')2 Fragment (Alexa Fluor® 555 Conjugate) (red) (1:1000) | #4413 | Cell Signaling Technology |
| Anti-Mouse IgG (H+L), F(ab')2 Fragment (Alexa Fluor® 488 Conjugate) (green) (1:1000) | #4408 | Cell Signaling Technology |
| (Alexa Fluor® 488-conjugated Goat Anti-Rabbit IgG (H+L)) | GB25303 | Servicebio |
| (Alexa Fluor® 594-conjugated Goat Anti-Mouse IgG (H+L) | GB28303 | Servicebio |

**Table S4. The critical commercial assays used in this study**

| Reagents | Catalog number | Source |
|---|---|---|
| Lenti-Pac HIV Expression Packaging Kit | LT002 | Genecopoeia |
| TB Green Premix Ex Taq II Kit | RR820A | Takara |
| Human VEGFA ELISA Kit | RAB0507 | Sigma-Aldrich |
| Human Angiopoietin-2 Quantikine ELISA Kit | DANG20 | R&D Systems |
| Duo-Luciferase HS Assay Kit | LF004 | GeneCopoeia |
| RIP Kit | Bes5101 | BersinBio |
| Epi$^{TM}$ ac4C immunoprecipitation kit | R1815 | Epibiotek |
| protein A/G magnetic beads | 8880210002D/10004D | Invitrogen |
| RNA pulldown Kit | Bes5102 | BersinBio |
| RNA Clean&Concentrator-5 kit | R1016 | ZYMO |
| Nuclear and Cytoplasmic Protein Extraction Kit | P0027 | Beyotime |
| VAHTS Stranded mRNA-seq Library Prep Kit | NR612-02 | Vazyme Biotech |
| Fluorescence in Situ Hybridization Kit | D-2968 | Exon Biotechnology Inc |
